# Modeling human pregastrulation development by 3D culture of blastoids generated from primed-to-naïve transitioning intermediates

**DOI:** 10.1093/procel/pwac041

**Published:** 2022-09-20

**Authors:** Zhifen Tu, Yan Bi, Xuehao Zhu, Wenqiang Liu, Jindian Hu, Li Wu, Tengyan Mao, Jianfeng Zhou, Hanwei Wang, Hong Wang, Shaorong Gao, Yixuan Wang

**Affiliations:** Translational Medical Center for Stem Cell Therapy & Institute for Regenerative Medicine, Shanghai East Hospital, School of Life Sciences and Technology, Tongji University, Shanghai 200120, China; Shanghai Key Laboratory of Maternal Fetal Medicine, Clinical and Translational Research Center of Shanghai First Maternity and Infant Hospital, School of Life Sciences and Technology, Tongji University, Shanghai 200092, China; Frontier Science Center for Stem Cell Research, Tongji University, Shanghai 200092, China; Translational Medical Center for Stem Cell Therapy & Institute for Regenerative Medicine, Shanghai East Hospital, School of Life Sciences and Technology, Tongji University, Shanghai 200120, China; Shanghai Key Laboratory of Maternal Fetal Medicine, Clinical and Translational Research Center of Shanghai First Maternity and Infant Hospital, School of Life Sciences and Technology, Tongji University, Shanghai 200092, China; Frontier Science Center for Stem Cell Research, Tongji University, Shanghai 200092, China; Translational Medical Center for Stem Cell Therapy & Institute for Regenerative Medicine, Shanghai East Hospital, School of Life Sciences and Technology, Tongji University, Shanghai 200120, China; Shanghai Key Laboratory of Maternal Fetal Medicine, Clinical and Translational Research Center of Shanghai First Maternity and Infant Hospital, School of Life Sciences and Technology, Tongji University, Shanghai 200092, China; Shanghai Key Laboratory of Maternal Fetal Medicine, Clinical and Translational Research Center of Shanghai First Maternity and Infant Hospital, School of Life Sciences and Technology, Tongji University, Shanghai 200092, China; Frontier Science Center for Stem Cell Research, Tongji University, Shanghai 200092, China; Translational Medical Center for Stem Cell Therapy & Institute for Regenerative Medicine, Shanghai East Hospital, School of Life Sciences and Technology, Tongji University, Shanghai 200120, China; Shanghai Key Laboratory of Maternal Fetal Medicine, Clinical and Translational Research Center of Shanghai First Maternity and Infant Hospital, School of Life Sciences and Technology, Tongji University, Shanghai 200092, China; Frontier Science Center for Stem Cell Research, Tongji University, Shanghai 200092, China; Shanghai Key Laboratory of Maternal Fetal Medicine, Clinical and Translational Research Center of Shanghai First Maternity and Infant Hospital, School of Life Sciences and Technology, Tongji University, Shanghai 200092, China; Frontier Science Center for Stem Cell Research, Tongji University, Shanghai 200092, China; Translational Medical Center for Stem Cell Therapy & Institute for Regenerative Medicine, Shanghai East Hospital, School of Life Sciences and Technology, Tongji University, Shanghai 200120, China; Shanghai Key Laboratory of Maternal Fetal Medicine, Clinical and Translational Research Center of Shanghai First Maternity and Infant Hospital, School of Life Sciences and Technology, Tongji University, Shanghai 200092, China; Frontier Science Center for Stem Cell Research, Tongji University, Shanghai 200092, China; Translational Medical Center for Stem Cell Therapy & Institute for Regenerative Medicine, Shanghai East Hospital, School of Life Sciences and Technology, Tongji University, Shanghai 200120, China; Shanghai Key Laboratory of Maternal Fetal Medicine, Clinical and Translational Research Center of Shanghai First Maternity and Infant Hospital, School of Life Sciences and Technology, Tongji University, Shanghai 200092, China; Frontier Science Center for Stem Cell Research, Tongji University, Shanghai 200092, China; Translational Medical Center for Stem Cell Therapy & Institute for Regenerative Medicine, Shanghai East Hospital, School of Life Sciences and Technology, Tongji University, Shanghai 200120, China; Shanghai Key Laboratory of Maternal Fetal Medicine, Clinical and Translational Research Center of Shanghai First Maternity and Infant Hospital, School of Life Sciences and Technology, Tongji University, Shanghai 200092, China; Frontier Science Center for Stem Cell Research, Tongji University, Shanghai 200092, China; Translational Medical Center for Stem Cell Therapy & Institute for Regenerative Medicine, Shanghai East Hospital, School of Life Sciences and Technology, Tongji University, Shanghai 200120, China; Shanghai Key Laboratory of Maternal Fetal Medicine, Clinical and Translational Research Center of Shanghai First Maternity and Infant Hospital, School of Life Sciences and Technology, Tongji University, Shanghai 200092, China; Frontier Science Center for Stem Cell Research, Tongji University, Shanghai 200092, China; Translational Medical Center for Stem Cell Therapy & Institute for Regenerative Medicine, Shanghai East Hospital, School of Life Sciences and Technology, Tongji University, Shanghai 200120, China; Shanghai Key Laboratory of Maternal Fetal Medicine, Clinical and Translational Research Center of Shanghai First Maternity and Infant Hospital, School of Life Sciences and Technology, Tongji University, Shanghai 200092, China; Frontier Science Center for Stem Cell Research, Tongji University, Shanghai 200092, China; Translational Medical Center for Stem Cell Therapy & Institute for Regenerative Medicine, Shanghai East Hospital, School of Life Sciences and Technology, Tongji University, Shanghai 200120, China; Frontier Science Center for Stem Cell Research, Tongji University, Shanghai 200092, China

**Keywords:** blastoids, primed, to, naïve conversion, transitioning intermediates, pregastrulation modeling

## Abstract

Human pluripotent stem cells provide an inexhaustible model to study human embryogenesis *in vitro*. Recent studies have provided diverse models to generate human blastoids by self-organization of different pluripotent stem cells or somatic reprogramming intermediates. However, whether blastoids can be generated from other cell types or whether they can recapitulate postimplantation development *in vitro* is unknown. Here, we develop a strategy to generate human blastoids from heterogeneous intermediates with epiblast, trophectoderm, and primitive endoderm signatures of the primed-to-naïve conversion process, which resemble natural blastocysts in morphological architecture, composition of cell lineages, transcriptome, and lineage differentiation potential. In addition, these blastoids reflect many features of human peri-implantation and pregastrulation development when further cultured in an *in vitro* 3D culture system. In summary, our study provides an alternative strategy to generate human blastoids and offers insights into human early embryogenesis by modeling peri- and postimplantation development *in vitro*.

## Introduction

Human embryogenesis initiates with a totipotent zygote, which finally develops into a mature individual. After fertilization of an oocyte, the zygote undergoes multiple cell cleavages to form a blastocyst, a process accompanied by zygotic genome activation (ZGA), cell polarization and morphogenesis, and lineage divergence into trophectoderm (TE) and inner cell mass (ICM) (Shahbazi, 2020). Subsequently, the blastocyst completes hatching from the zona pellucida and starts implantation at E6 (Shahbazi and Zernicka-Goetz, 2018). During implantation, ICM cells undergo fate specification and develop into epiblasts (EPIs) and primitive endoderm (PrE, also known as hypoblasts), which are characterized by high expression of NANOG and GATA6, respectively (Nakamura et al., 2016; Wu and Izpisua Belmonte, 2016; Shahbazi et al., 2017). Notably, EPI cells transition from an unrestricted state of naïve pluripotency toward an ‘onset of differentiation’ state of primed pluripotency during the implantation process (Rossant and Tam, 2017; Yilmaz and Benvenisty, 2019), followed by gastrulation and ultimately the formation of all cell types of the body. PrE and TE cells further differentiate into various extraembryonic cell types and eventually give rise to extraembryonic yolk sac and placenta, respectively, providing nutrition and support for the fetus (Rossant and Tam, 2017).

The development from a single zygote to a mature individual with more than 200 cell types depends on the complicated and orderly regulation of embryogenesis. However, the scarcity of embryo resources, the difficulty of embryo culture *in vitro*, and the constraints of ethical policies have greatly limited research on human embryogenesis. Multiple types of pluripotent stem cell (PSC) lines either derived from blastocysts or reprogrammed from somatic cells have been used to construct blastocyst-like structures (termed blastoids), offering inexhaustible resources for embryogenesis modeling (Fu et al., 2021; Rossant and Tam, 2021). Successful trials have been made to generate blastoids using embryonic stem cells (ESCs) and extended pluripotent stem cells (EPSCs) in mice (Bedzhov and Zernicka-Goetz, 2014; Harrison et al., 2017; Beccari et al., 2018; Rivron et al., 2018; Sozen et al., 2018, 2019; Li et al., 2019; Zhang et al., 2019; Veenvliet et al., 2020). Recent studies have reported that human blastoids can be generated from ESCs at the naïve pluripotent state, naïve reprogramming intermediates, and EPSCs under certain conditions (Fan et al., 2021; Liu et al., 2021; Sozen et al., 2021; Yanagida et al., 2021; Yu et al., 2021; Kagawa et al., 2022). These blastoids, which share similarities with natural embryos in many aspects, including morphology, lineage composition and localization, and transcriptome, can serve as invaluable models for mimicking human peri- and early postimplantation development *in vitro*.

Our recent study charting the cell fate roadmap showed the appearance of EPI, TE, and PrE signatures in the heterogeneous populations of cells transitioning from a primed state to naïve pluripotency (Bi et al., 2022). Here, we describe a strategy to generate human blastoids by *in vitro* self-organization from the transitioning intermediates of the primed-to-naïve conversion process. Characterization of these blastoids reveals that they resemble human blastocysts in many aspects, including morphology, size dimension, architecture of cell lineages, and the capacity to derive embryonic and extraembryonic stem cell lines. scRNA-seq analysis further confirmed their transcriptomic similarity to natural blastocysts. In addition, the 2D culture of blastoids can model several characteristics of human embryos at the early stages of peri-implantation. A recently developed 3D culture system drives these blastoids to further develop into pregastrulation-like structures, recapitulating key events that occur during the early stages of postimplantation *in vitro*. Taken together, coupled with the *in vitro* 3D culture conditions and the primed-to-naïve conversion system, our study provides an alternative strategy to generate human blastoids and model early embryogenesis up to the pregastrulation stage, facilitating insights into human embryonic development.

## Results

### Generation of human blastoids using the transitioning cells of the primed-to-naïve conversion process

We recently reported the appearance of EPI, TE, and PrE signatures in the transitioning populations of cells from the primed state to naïve pluripotency by charting the cell fate map using a dual fluorescent reporting system composed of *ALPG*-promoter-RFP and *OCT4*-ΔPE-GFP (Bi et al., 2022). Briefly, on day 6 during the primed-to-naïve transition process, scoring analysis of the single-cell RNA sequencing (scRNA-seq) datasets revealed the emergence of strong EPI, TE, and PrE signatures in different subpopulations of transitioning intermediates (Fig. S1A). Likewise, immunoﬂuorescence labeling also showed the existence of OCT4-, CDX2-, and GATA6-positive cell subpopulations (Fig. S1B). Interestingly, the OCT4-expressing cells clustered as colonies with morphologies similar to naïve PSCs, and the cells expressing CDX2 or GATA6 either surrounded or scattered outside these OCT4-positive colonies (Fig. S1B). Similar results could also be observed in the transitioning cells on day 8 toward naïve pluripotency from H9 human ESCs (hESCs) either engineered with the *ALPG*-promoter-RFP (APR) reporter or not (Fig. S1C–E). Notably, the RFP signals of the *ALPG*-promoter reporter coincided with the OCT4 staining signals in the transitioning cells with the APR reporter (Fig. S1D). However, cell ratio counting revealed higher proportions of TE-like (CDX2-positive) or PrE-like (GATA6-positive) subpopulations on day 8 than on day 6 (Fig. S1F), suggesting that the heterogeneous intermediate cells on day 8 of the primed-to-naïve conversion process may be adopted as a source for human blastoid modeling.

**Figure 1.**
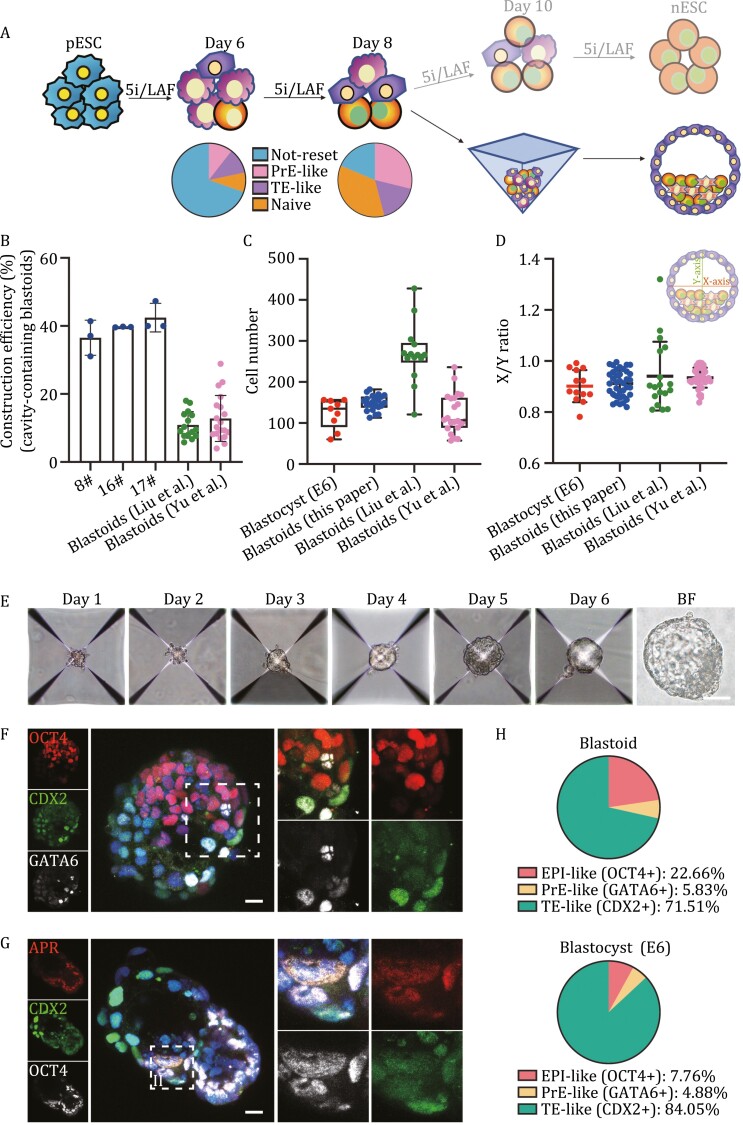
Generation of human blastoids from the transitioning intermediate cells of the primed-to-naïve transition process. (A) Schematic of human blastoid generation from the intermediate cells of the primed-to-naïve transition process. (B) Efficiencies of cavity-containing structure formation from intermediates of the primed-to-naïve transition process using different protocols shown in Fig. S2A as well as published blastoids (data from Liu et al., 2021; Yu et al., 2021); mean ± SD. (C) Total number of cells per human blastocyst (*n* = 9 biological replicates), human blastoid (*n* = 22 biological replicates), and published blastoids (data from Liu et al., 2021; Yu et al., 2021). Box plots show the median (center line), 25th and 75th percentiles (bottom and top of box, respectively), and minimum and maximum values (bottom and top whisker, respectively). (D) Measurement of the *x*/*y* ratio of blastoids (*n* = 38) and published blastoids (data from Liu et al., 2021; Yu et al., 2021), compared to blastocysts (*n* = 13) (data for blastocysts from Liu et al., 2021); mean ± SEM. (E) Representative phase-contrast images of cell aggregates at the indicated time points during blastoid formation. Scale bar, 100 μm. (F) Representative coimmunostaining images of OCT4, CDX2 and GATA6 in a human blastoid. Higher magnification images of selected planes of the boxed areas are shown on the right. Scale bars, 20 μm. (G) Representative coimmunostaining images of CDX2 and OCT4 in a human blastoid generated from intermediate cells during naïve pluripotency establishment from pESCs integrated with the ALPG-promoter-RFP reporter. Higher magnification images of selected planes of the boxed areas are shown on the right. Scale bars, 20 μm. (H) Pie charts showing the frequencies of EPI-like, TE-like, and PrE-like cells in human blastoids (upper) and human blastocysts (bottom) indicated by expression patterns of OCT4, CDX2, and GATA6, respectively.

Next, we harvested the intermediate cells of H9 hESCs on day 8 toward naïve pluripotency that contained 35.5% EPI-like, 17.0% TE-like, 28.8% PrE-like subpopulations and 18.62% non-reset cells, and transferred them into AggreWell plates fed with blastoid induction medium to generate blastoids (Fig. 1A, see Methods for details). We tried different induction conditions to generate blastoid structures morphologically resembling human blastocysts at E6 by adjusting the proportions of components of the medium or the numbers of starting intermediate cells. Mixing IVC1: naïve medium:TSM at ratios of 2:1:1, 4:1:1, and 1:1:1 resulted in the highest efficiencies (~40%) in forming cavity-containing structures among all the conditions (Fig. 1B, see Methods for details), which is also higher than recently published protocols developed by Wu group or Polo group (Liu et al., 2021; Yu et al., 2021), while cells cultured in other conditions failed due to small or no cavity formation (Fig. S2A). We also found that induction starting with ~7 × 10^4^ cells per well could generate blastoids with overall size and dimensions similar to those of natural human embryos at E6 (Fig. 1C and 1D). Thus, we successfully produced human blastoids from the primed-to-naïve transitioning intermediates under the optimized induction conditions (starting with ~7 × 10^4^ cells and culturing in IVC1:naïve medium:TSM at 2:1:1). Similar to the blastoids generated by other protocols (Liu et al., 2021), cells aggregated after 24 h of induction and formed small cavity-containing structures on day 3 or day 4, which exhibited typical blastocyst-like structures by day 6, with a visibly enlarged cavity (Figs. 1E and S2B).

Next, we characterized the EPI, TE, and PrE lineages of the blastoids derived from day 8 intermediate cells of the primed-to-naïve conversion as described above. Immunofluorescence staining results revealed that OCT4-positive cells localized in the inner cell layers of the blastoid structure (Fig. 1F and 1G), whereas CDX2-positive cells representing TE signatures were located in the outer cell layer surrounding the OCT4-positive cells (Fig. 1F and 1G), resembling the specification of TE and ICM in natural blastocysts at E6 (Fig. S2C). In addition, when using the cells integrated with the APR reporter for blastoid generation, RFP-positive cells also specifically indicated ICM-like cells of the blastoids with strong OCT4 expression, which were surrounded by CDX2-positive cells with strong TE signatures (Fig. 1G), consistent with the staining results of lineage markers in day 8 intermediate cells. We also quantified the ratios of OCT4-positive (EPI-like), CDX2-positive (TE-like), and GATA6-positive (PrE-like) cells in natural blastocysts and blastoids, respectively, and found that these constructed blastoids contained more EPI-like cells and fewer TE-like cells than natural blastocysts (Fig. 1H). In addition, we also generated abnormal blastoids with small or no cavity structures (Fig. S2D).

In contrast to the outer layer cells expressing CDX2, GATA6-positive cells were less abundant and scattered in the TE-like layer or ICM-like structure of the blastoids (Fig. 1F and 1G). A small proportion of these GATA6-positive cells also showed strong expression of CDX2 or OCT4 (Fig. 1F), similar to previous observations in natural blastocysts at E6 (Xiang et al., 2020). We also labeled membrane contours to visualize the cellular morphologies of the blastoids with F-actin antibodies. While the outer cells that formed the cavity-like structure were flat, the NANOG-positive cells that were clustered as the ICM-like structure exhibited round cell contours (Fig. S2E). Moreover, we observed laminin expression in both EPI-like and TE-like cells of the blastoid (Fig. S2F). Taken together, these immunostaining results showed that the human blastoids derived from the day 8 intermediates of the primed-to-naïve transition possess a three-layer structure with EPI, TE, and PrE signatures resembling those of natural blastocysts.

### Transcriptional profiling of blastoids

To further assess the transcriptional states of the cells in human blastoids generated from the day 8 intermediates during the primed-to-naïve transition, we performed scRNA-seq analysis on 1,190 cells obtained from these blastoids (Fig. S3A). Signature scoring analysis showed the presence of EPI-like, TE-like, and PrE-like populations in the blastoids with all cells characterized (Fig. S3A) according to the corresponding gene signatures and distribution. However, we observed that EPI-like cells were more than TE-like cells in the blastoids (Fig. S3A), which is different from natural blastocysts at E5–E7 (Petropoulos et al., 2016).

**Figure 2.**
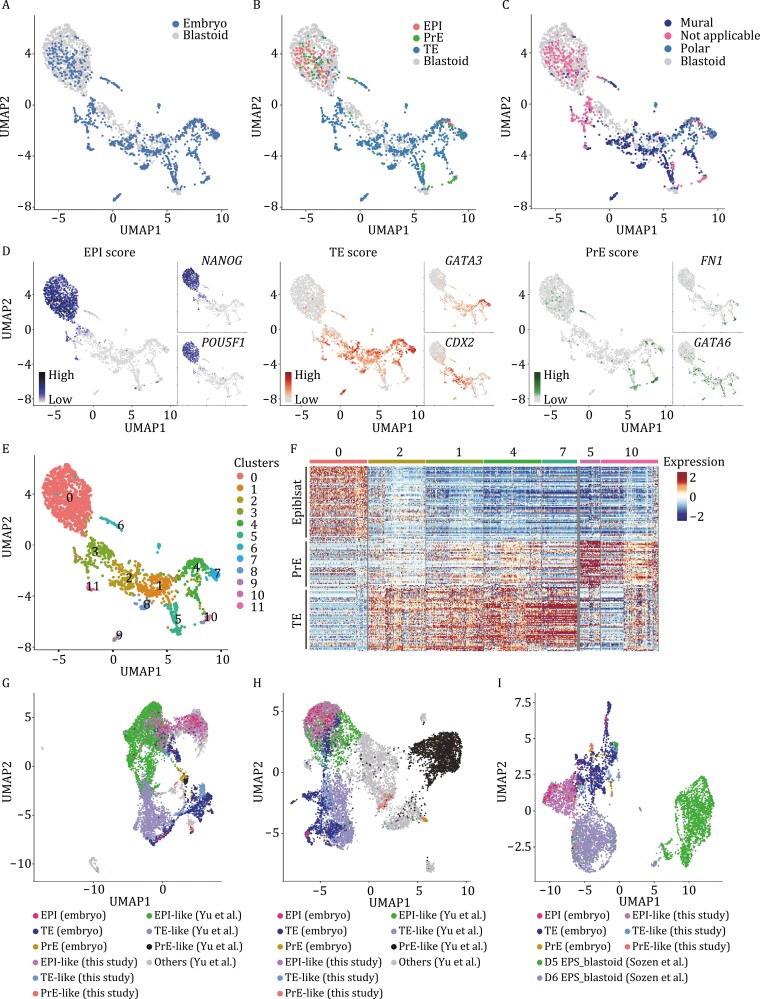
Single-cell transcriptional profiling of human blastoids. (A) UMAP plots of the integrated scRNA-seq datasets (a total of 2286 cells) from blastoids (gray) and human preimplantation embryos (Petropoulos et al., 2016) (blue). (B) UMAP plots highlighting cells of blastoids (gray) and cells derived from human preimplantation embryos with EPI (red), TE (blue), and PrE (green) signatures. (C) UMAP plots highlighting cells of blastoids (gray) and cells derived from human preimplantation embryos with mural (dark blue) and polar (light blue) TE signatures. (D) Per-cell expression score for EPI, TE, and PE signatures of the blastoid scRNA-seq dataset integrated with human preimplantation embryo scRNA-seq datasets (Petropoulos et al., 2016) (left). Representative EPI (*POU5F1* and *NANOG*), TE (*CDX2* and *GATA3*), and PrE (*FN1* and *GATA6*) marker expression on UMAP (right). (E) Cell clustering projection on UMAP; total of 12 clusters. (F) Heat map showing the EPI, TE, and PrE signatures in the different clusters shown in Fig. 2E. (G) UMAP embedding of single-cell transcriptomes from blastoids, human preimplantation embryos (Petropoulos et al., 2016), and stem blastoids (Yu et al., 2021). (H) UMAP embedding of single-cell transcriptomes from blastoids, human preimplantation embryos (Petropoulos et al., 2016), and iblastoids (Liu et al., 2021). (I) UMAP embedding of single-cell transcriptomes from blastoids, human preimplantation embryos (Petropoulos et al., 2016), and hEPSC structures (Sozen et al., 2021).

To further characterize the blastoids, we performed an integrated analysis with published scRNA-seq data derived from human preimplantation embryos at E5–E7 (Petropoulos et al., 2016) to compare the transcriptional similarities between blastoids and natural blastocysts. UMAP analyses revealed that the blastoid cells clustered with the cells derived from human blastocysts (Fig. 2A), especially the E5–E6 embryos (Fig. S3B). In addition, the EPI-, TE-, and PrE-like clusters in the blastoids coincided with the lineage counterparts in the natural blastocysts across developmental time (Figs. 2B and S3C). Moreover, we observed two distinct subpopulations in the TE-like cells of blastoids, which show high concordance with polar TE cells and mural TE cells (Fig. 2C), as has been reported in human blastocysts (Petropoulos et al., 2016).

We also identified blastocyst lineages in the blastoid scRNA-seq dataset that overlapped with the published transcriptome of human blastocysts. Scoring analyses exhibited EPI, TE, and PrE signatures in respective clusters on UMAP, which were further confirmed by specific expression of marker genes of different lineages (*NANOG* and *POU5F1* for EPI, *GATA3* and *CDX2* for TE, and *FN1* and *GATA6* for PrE) (Fig. 2D). Using unsupervised clustering analysis, we further identified 12 cell clusters (Fig. 2E), in which we characterized clusters with EPI signatures (Cluster 0), PrE signatures (Clusters 5 and 10), and TE signatures (Clusters 1, 2, 4, and 7) according to the expression of corresponding marker genes (Fig. 2F). Interestingly, we also observed strong and specific expression of polar TE marker genes such as *MUC15* and *OVOL1* in cluster 4 and cluster 7 (Fig. S3D), suggesting the presence of polar TE-like subpopulations among the TE-like identities.

Next, to compare our blastoids with the published blastoids generated from different types of starting cells at the transcriptional level, we performed an integrative analysis with stem blastoids derived from naïve hPSCs (Yu et al., 2021), iblastoids derived from human somatic reprogramming intermediates toward naïve pluripotency (Liu et al., 2021), and cystic structures derived from human extended pluripotent stem cells (hEPSCs) (Sozen et al., 2021), respectively. Analyzing the lineage compositions of these blastoid datasets, we found that all published blastoids contain large proportions of undefined clusters, except for blastoids generated in this study with all cells characterized and defined into EPI-, TE-, or PrE-like lineages (Figs. 2G–2I and S3E). Integrative UMAP plots showed that all EPI-like cells of blastoids were well correlated with EPI cells from natural blastocyst, except for hEPSC-derived structures, which showed little alignment with blastocyst (Fig. 2G–I). Additionally, we noted that the TE-like cells of the blastoids we generated were more concordant with the TE cells of natural blastocysts than those of other blastoids. We then investigated the expression of representative EPI-specific genes (*POU5F1*, *NANOG*), TE-specific genes (*GATA3*, *CDX2*), and PrE-specific genes (*FN1*, *PDGFRA*) among these blastoids. The blastoids we produced exhibited similar expression patterns of lineage-specific genes to natural blastocysts, and more specific compared to other blastoids (Fig. S4A–C).

We also examined the expression of representative amnion-specific genes (*ISL1* and *GABRP*) among blastoids generated from different studies. Different from the TE-like cells from iblastoids that highly expressed amnion-specific genes, both the stem blastoids and blastoids we produced showed low expression of these genes in the TE-like cells, similar to natural blastocysts (Fig. S4D), consistent with the observation as has been reported that the TE counterpart of iBlastoids generated from human somatic reprogramming intermediates may share properties with the amnion rather than TE (Zhao et al., 2021).

Taken together, the transcriptional results suggest great similarities between blastoids we produced and natural blastocysts, including lineage composition, specification of TE, lineage cells alignment, and genes expression patterns.

### Derivation of stem cell lines

**Figure 3.**
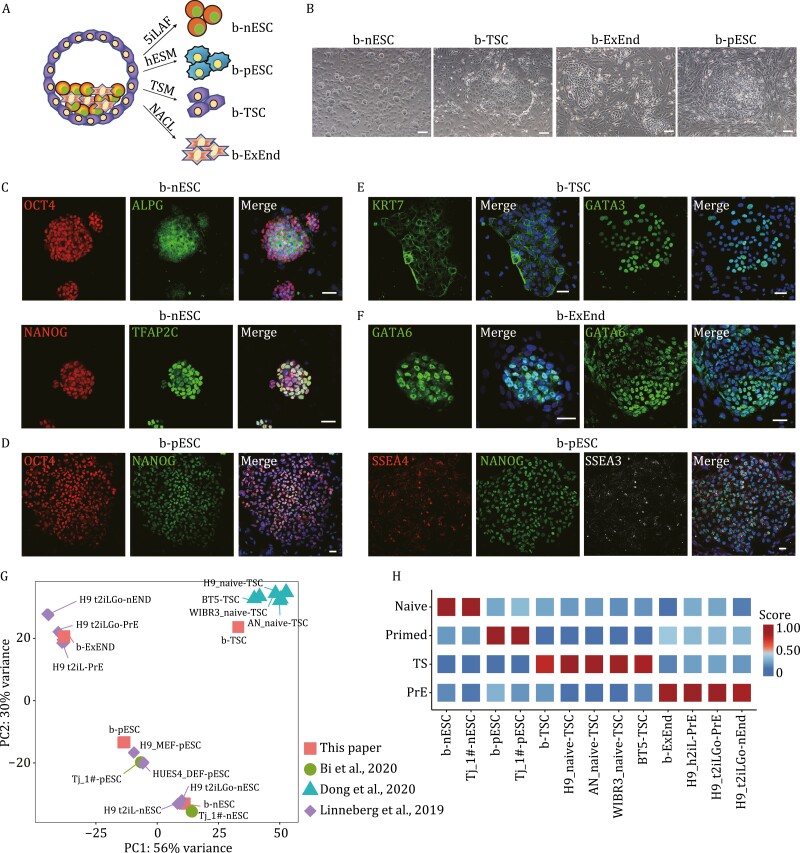
Derivation of different types of stem cells from human blastoids. (A) Experimental design for the derivation of b-nESCs, b-pESCs, b-TSCs and b-ExEnd cells from blastoids. (B) Representative phase contrast of b-nESCs, b-pESCs, b-TSCs, and b-ExEnd cells. Scale bars, 100 μm. (C–F) Immunofluorescence images of b-nESCs (C), b-pESCs (D), b-TSCs (E), and b-ExEnd cells (F) stained with representative markers. Scale bars, 50 μm. (G) PCA of the bulk RNA-seq datasets from blastoid-derived cell lines with published RNA-seq datasets (Linneberg-Agerholm et al., 2019; Bi et al., 2020; Dong et al., 2020). *n *≥ 2. (H) Naïve pluripotency, primed pluripotency, TSC, and PrE signature scores of the blastoid-derived cell lines with published PSC lines.

ESCs and trophoblast stem cells (TSCs) can be derived from natural blastocysts *in vitro*. Similarly, following 5iLAF (Theunissen et al., 2014), hESC (Yu et al., 2007), TSC (Okae et al., 2018), and NACL (Linneberg-Agerholm et al., 2019) culture conditions reported previously, we successfully derived naïve ESCs, primed ESCs, TSCs, and extraembryonic endoderm (ExEnd) cell lines from individually plated blastoids (referred to as b-nESCs, b-pESCs, b-TSCs, and b-ExEnd cells, respectively) (Figs. 3A and S5A). All these cell lines exhibited typical morphologies similar to those derived from natural blastocysts and could be maintained for at least 20 passages (Fig. 3B). Using immunofluorescence, we detected robust expression of epiblast-specific markers, including ALPG, NANOG, OCT4, and TFAP2C, in b-nESCs (Fig. 3C); strong expression of the primed state-specific surface markers SSEA3 and SSEA4, as well as the pluripotent markers OCT4 and NANOG, in b-pESCs (Fig. 3D); specific expression of the trophoblast markers KRT7 and GATA3 in b-TSCs (Fig. 3E); and notable expression of the hypoblast marker GATA6 in b-ExEnd cells (Fig. 3F). In addition, bulk RNA-seq analysis revealed that while the blastoid-derived cell lines segregated into distinct groups, each group clustered with the corresponding published cell lines (Fig. 3G) (Linneberg-Agerholm, et al., 2019; Bi et al., 2020; Dong et al., 2020). As expected, scoring analysis also confirmed different signatures, including naïve and primed pluripotent state signatures, TSC signatures and PrE signatures, in the corresponding blastoid-derived cell lines (Fig. 3H). Altogether, these results indicate that human blastoids are capable of deriving stem cell lines with blastocyst lineage identities.

We also subjected some of these stem cell lines to differentiation to assess their developmental potentials. By embryoid body formation assays (Fig. S5B), b-pESCs could differentiate into cells from all three germ layers based on the specific expression of E-CADHERIN and NESTIN (ectoderm markers), BRACHYURY (a mesoderm marker), and GATA6 and SOX17 (endoderm markers) (Fig. S5C). Moreover, b-TSCs were successfully differentiated into syncytiotrophoblast (ST)-like cells, as observed by immunofluorescence of positive staining for CGB and SDC1 (Fig. S5D). Collectively, these data demonstrate that the stem cell lines derived from blastoids possess differentiation capacities *in vitro*.

### 2D culture of blastoids for peri-implantation modeling *in vitro*

**Figure 4.**
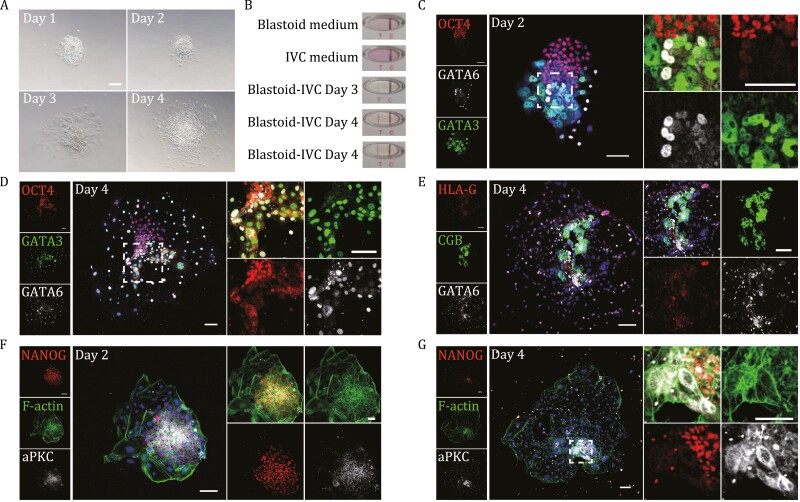
Modeling peri-implantation by 2D culture of the blastoids. (A) Representative bright-field images of attached blastoids on days 1–4 of 2D *in vitro* culture. Scale bars, 100 μm. (B) Representative results of the hCG test on medium collected from day 3 and day 4 attached blastoids using a human pregnancy test kit; blastoid culture medium and IVC medium were used as controls. (C) Representative coimmunostaining images of OCT4, GATA3 and GATA6 in a day 2–attached human blastoid. Scale bars, 100 μm. (D) Representative coimmunostaining images of OCT4, GATA3, and GATA6 in a day 4–attached human blastoid. Scale bars, 100 μm. (E) Representative coimmunostaining images of HLA-G, CGB, and GATA6 in a day 4–attached human blastoid. Scale bars, 100 μm. (F) Representative coimmunostaining images of NANOG, F-actin and aPKC in a day 2–attached human blastoid. Scale bars, 100 μm. (G) Representative coimmunostaining images of NANOG, F-actin, and aPKC in a day 4–attached human blastoid. Scale bars, 100 μm.

To evaluate whether further *in vitro* culture can drive the self-organization of these blastoids into peri-implantation or postimplantation embryo-like structures, we adopted an *in vitro* attachment assay reported previously (Deglincerti et al., 2016; Shahbazi et al., 2016) and performed 2D culture of blastoids to monitor their morphological changes for an additional 4 days in IVC medium. Approximately 80% of blastoids attached to the dish within 1 day (Fig. 4A). After attachment, the blastoids flattened, expanded, and progressed to form outgrowth structures resembling the changes observed in human blastocysts as well as blastoids derived by other protocols cultured in IVC (Shahbazi et al., 2016) (Fig. 4A). We also detected an increased level of human chorionic gonadotrophin (hCG) secretion in the IVC medium of attached blastoids since day 3 of the *in vitro* culture, while no hCG secretion could be observed in blank IVC medium or blastoid induction medium after 4 days of culture of blastoids (Fig. 4B), suggesting successful transition from TE-like cells of the blastoids to ST-like cells *in vitro*.

On day 2 of IVC culture, the number of cells expressing OCT4, CDX2, and GATA6 increased (Fig. 4C), indicating that cells of the blastocyst lineages expanded and spread after attachment. In addition, lineage segregation could also be observed in the outgrowth (Figs. 4C and S6A). EPI-like cells marked by OCT4 or NANOG expression were located in the center and were encircled by GATA6- and GATA3-positive cells representing the PrE and TE lineages, respectively (Figs. 4C and S6A). In particular, most PrE-like cells with GATA6 expression were located close to the peri-edge of the EPI-like structures in the outgrowth (Figs. 4C and S6A). However, a small proportion of PrE-like cells were also positive for the TE lineage marker GATA3 (Figs. 4C and S6A). Distinct segregations among the three lineages could not be observed until day 4 in IVC culture (Figs. 4D and S6B), consistent with the observations in natural blastocysts (Shahbazi et al., 2016). We also observed dramatically decreased expression of CDX2 in the TE-like cells of blastoids upon attachment (Fig. S6C), which indicates differentiation of the TE-like cells to trophoblast-like cells during the 2D culture. Beyond hCG secretion as mentioned above and CGB expression by immunofluorescence, immunostaining of HLA-G in day 4–attached blastoids further confirmed the *in vitro* differentiation of the trophoblast-like lineage into not only ST-like cells but also extravillous cytotrophoblast (EVT)-like cells (Fig. 4B and 4E). Moreover, we also observed specific expression of aPKC within the Nanog-positive EPI-like compartment of the attached blastoids as early as day 2; however, there were no cavity-like structures (Fig. 4F). On day 4 of IVC culture, costaining for F-actin, NANOG, and aPKC revealed the formation of a proamniotic cavity-like structure in the attached blastoid, as previously reported (Fig. 4G) (Liu et al., 2021). Furthermore, a primary yolk sac–like structure could also be observed by immunostaining of GATA6 at that time (Fig. S6D). In addition, the presence of multinucleation observed in outer cells of day 4–attached blastoids further confirmed the differentiation of TE-like cells into ST-like cells (Fig. 4G). Taken together, the 2D IVC culture of the blastoids generated from transitioning intermediates of the primed-to-naïve conversion process recapitulated some features of peri-implantation development of natural blastocysts *in vitro*.

### 3D culture of blastoids for pregastrulation modeling *in vitro*

**Figure 5.**
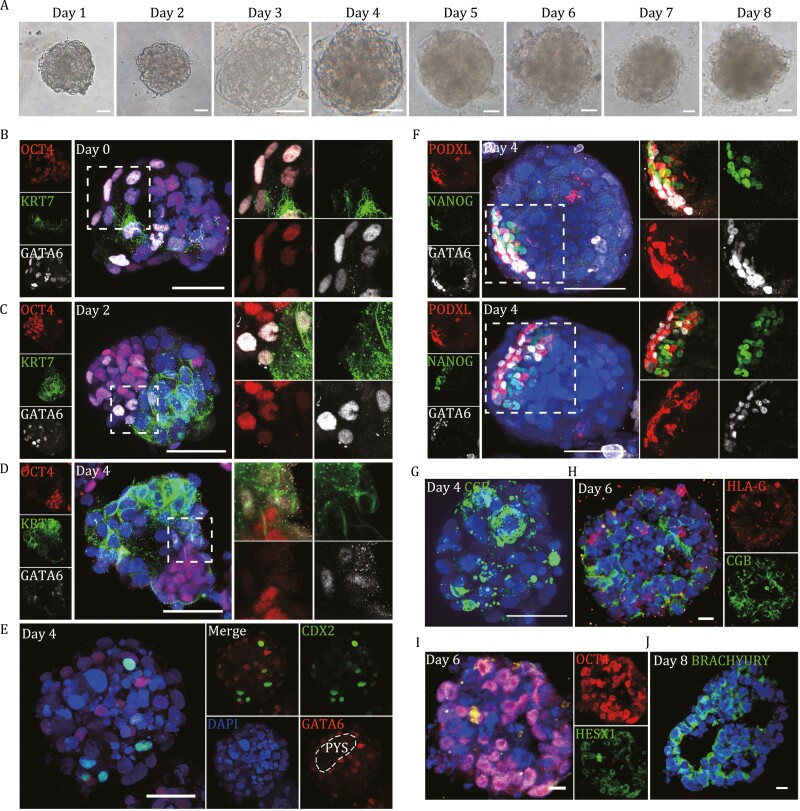
Modeling human postimplantation development by 3D culture of blastoids. (A) Representative bright-field images of human blastoids from day 1 to day 8 cultured in the 3D system developed for *in vitro* culture of blastocysts (Xiang et al., 2020). Scale bars, 100 μm. (B–D) Representative coimmunostaining images of OCT4, KRT7, and GATA6 in the blastoids cultured in the 3D system on day 0 (B), Scale bars, 50 μm; day 2 (C) and day 4 (D), Scale bars, 100 μm. (E) Representative coimmunostaining images of CDX2 and GATA6 in a blastoid cultured in the 3D system on day 4. Scale bars, 100 μm. Dashed lines indicate the PYS area. (F) Representative coimmunostaining images of NANOG, GATA6 and PODXL in blastoids cultured in the 3D system on day 4. Scale bars, 100 μm. (G) Representative immunostaining images of CGB in a blastoid cultured in the 3D system on day 4. Scale bars, 50 μm. (H) Representative coimmunostaining images of HLA-G and CGB in a blastoid cultured in the 3D system on day 6. Scale bars, 20 μm. (I) Representative coimmunostaining images of OCT4 and HESX1 in a blastoid cultured in the 3D system on day 6. Scale bars, 20 μm. (J) Representative immunostaining images of BRACHYURY in a blastoid cultured in the 3D system on day 8. Scale bars, 20 μm.

We further cultured the blastoids in a 3D culture system to mimic human pregastrulation development following a recently established protocol. Continuous morphological observations shifted from 1 to 8 days revealed a stepwise developmental progression (Fig. 5A), corresponding to that of human blastocysts developing from 7 to 14 days postfertilization (d.p.f.) *in vitro* as reported (Xiang et al., 2020). Interestingly, while the expression of the epiblast marker OCT4, the TE marker CDX2, and the hypoblast marker GATA6 could be observed in the blastoids on day 6 of induction (Fig. 1E), the expression of KRT7, the trophoblast marker, was rarely detected (Figs. 5B and S7A). Upon 3D culture, the number of CDX2-positive cells was significantly decreased and was nearly undetectable on day 4 of the 3D culture (Fig. S7B), similar to our observations of the attached blastoids in the 2D culture. However, the expression of KRT7 was greatly increased (Fig. 5C and 5D), again suggesting a cell fate transition from a TE-like to a trophoblast-like state. We also noticed an increase in mutually exclusive expression patterns of OCT4, GATA6, and KRT7 in the 3D culture of blastoids since day 2 (Fig. 5C and 5D), suggesting the gradual polarization and reorganization of the epiblast-like cells and increased specificity among the derivatives of the three lineages (Fig. 5C and 5D), as observed in natural human embryos (Xiang et al., 2020). On day 4 of the 3D culture, we observed the PYS-like structure by the presence and distribution of GATA6-positive cells (Fig. 5E), coincident with the observations in *in vitro* cultured human embryos at E10 (Xiang et al., 2020). At that time, EPI-like cells marked by NANOG expression polarized and rearranged radially (Figs. 5F and S7C). The radial expression pattern of podocalyxin (PODXL) and the orderly arrangement of GATA6-positive cells surrounding NANOG-positive cells also indicated polarization and epithelialization in the EPI-like cell population (Figs. 5F and S7C). Moreover, CGB was robustly expressed and localized in the outer cell layer of the 3D-cultured blastoids (Figs. 5G, 5H, S7D, and S7E), suggesting successful differentiation of the trophoblast-like cells. On day 6, we also detected the expression of the EPI marker HESX1 (Figs. 5I and S7F), which represents the differentiation of early anterior or visceral endoderm, while the expression of BRACHYURY, a marker of the primitive streak, could not be detected until day 8 of 3D culture (Fig. 5J). Taken together, the observations above indicated that blastoids could be used to model human postimplantation development before gastrulation *in vitro*.

## Discussion

In this study, we successfully developed a strategy to generate human blastoids from intermediate cells of the primed-to-naïve transition process. Different from the recently published study that adopted the somatic reprogramming intermediates for blastoid construction (Liu et al., 2021), our study relies on the primed-to-naïve transition system which excludes the introduction of exogenous OSKM genes, thus generating transgene-free blastoids. Besides, cells within blastoids generated in our study can be well characterized into three blastocyst lineages without undefined clusters or amnion signal (Figs. S3E and S4D). The undefined clusters within stem blastoid and iblastoid contain substantial numbers of mesoderm-like cells (Zhao et al., 2021), suggesting features in line with postimplantation human embryos. Additionally, blastoids generated in this study show great similarities with natural human blastocysts in terms of morphological architectures, gene expression patterns, and lineage differentiation potentials. More importantly, these human blastoids exhibit many characteristics resembling natural embryogenesis up to pregastrulation when further cultured in an *in vitro* 3D culture system developed recently. These results collectively present an alternative *in vitro* model to mimic human early embryonic development using primed-to-naïve transitioning intermediates.

Our blastoid construction strategy depends on the intermediate cells collected from the primed-to-naïve state transition process, and these heterogeneous cells could reduce the inference of starting cell conditions to some extent. In addition, systematic comparison with other blastoids generated from different types of starting cells as well as natural blastocysts revealed improved production efficiency and well-characterized cell lineages without undefined clusters or amnion signals in blastoids produced by our strategy (Table 1). We also compared the stem cell line derivation capacities and developmental landscapes upon further *in vitro* cultures of blastoids generated by different protocols with natural blastocysts (Table 1). Notably, blastoids generated in this study exhibit similar and synchronous developmental progression to natural blastocysts during their further culture in the 3D culture system (Xiang et al., 2020), including expansion and polarity of EPI-like cells, differentiation of TE-like and PrE-like counterparts, and emergence of primitive streak features (Table 1 and Fig. 5). However, limitations were also observed in our blastoid model. For example, while our induction system leads to efficient cavity formation (~40%) during self-organization of blastoids, the immunostaining results indicate that many blastoids exhibit structural abnormalities compared to natural blastocysts, with an incorrect composition or allocation of TE and EPI lineages. In addition, similar as other blastoids generated by different strategies, the blastoids we generated showed an incorrect proportion of cell lineages, compared to natural blastocysts (Fig. S3E and Table 1). scRNA-seq analysis also revealed that the blastoids generated from transitioning cells of the primed-to-naïve transition still possess a much greater EPI-like population than the TE-like population, different from the natural human blastocysts, indicating further optimizations of blastoid derivation are still needed.

**Table 1.** Summary of protocols to generate blastoids from different types of starting cells.

	Culture medium		E6 embryo	This study	Liu et al.	Yu et al.	Sozen et al.
Induction system	Starting cells		-	Primed-to-naïve intermediates	Somatic reprograming intermediates	Naïve hPSCs	EPSCs
	Starting cells numbers		-	7 × 10^4^	9 × 10^4^	3 × 10^4^	0.72 × 10^4^
	Induction timing		-	8 + 6 d	21 + 6 d	9 d	5 d
	Construction efficiency (cavity-containing blastoids)		-	~40%	5.8%–18%	4%–28.8%	~7%
Blatoids features	Cell numbers		~123	~149	~269	~121	Lack of primary data
	Blastoids diameter		0.902	0.916	0.94	0.941	Lack of primary data
	Lineage proportion (%)	EPI	7.76	22.66	15.4	14.46	Lack of primary data
		PrE	4.88	5.83	9.8	3.89	Lack of primary data
		TE	84.05	71.51	81.8	78.11	Lack of primary data
	Stem cell line derivation capacity	Naïve ESC	Yes	Yes	Yes	Yes	-
		TSC	Yes	Yes	Yes	Yes	-
		End	Yes	Yes	-	Yes	-
		Primed ESC	Yes	Yes	Yes	-	-
	Development potential	Epi expansion	Yes	Yes	Yes	Yes	-
		Polarity	Yes	Yes	-	-	-
		TE differentiation	Yes	Yes	Yes	Yes	-
		PYS formation	Yes	Yes	-	Yes	-
		Primitive streak feature	Yes	Yes	-	-	-
Transcriptome	Amnion feature		None	None	Yes	None	Yes
	Proportion of lineages (%)	EPI	10.843	65.042	16.914	33.192	7.045
		TE	79.759	29.327	20.647	21.301	3.571
		PrE	9.397	5.630	24.190	0.785	28.669
		Undefined	0	0	38.247	44.721	60.714

To model embryogenesis *in vitro*, we not only used conventional 2D attachment assays but also adopted the recently established 3D culture system with Matrigel. Using these culture conditions, we described the characteristics of these embryo-like structures that simulate early embryonic development from peri-implantation to the pregastrulation stage, including the emergence of PYS, trophoblast differentiation, and the expression of the primitive streak marker T. However, the efficiency of the *in vitro* 3D culture of blastoids decreases rapidly with time, indicating that further optimization of the *in vitro* culture system is still needed.

In conclusion, our study provides an alternative and efficient strategy for human embryogenesis modeling. Combined with gene editing and drug screening, our study will shed light on both basic and translational research on human embryogenesis and early embryonic defects in the future.

## Materials and methods

### Cell culture

Human primed ESCs were propagated in a conventional hESC medium consisting of DMEM/F-12 (Thermo Fisher) with 20% KnockOut SR (Thermo Fisher), 1% nonessential amino acids (Millipore), 2 mmol/L GlutaMAX (Millipore), penicillin-streptomycin (Millipore), and 8 ng/mL bFGF (PeproTech). The cells were passaged every 5–6 days using 0.5 mmol/L EDTA onto mitotically inactivated mouse embryonic fibroblast (iMEF) feeders, with the medium changed daily. Mycoplasma tests were routinely conducted. Naïve ESCs were cultured in 5iLAF medium containing DMEM/F-12:Neurobasal (1:1) (Thermo Fisher), 1% N2 supplement (Thermo Fisher), 2% B27 supplement (Thermo Fisher), 0.5% KnockOut SR (Thermo Fisher), 1% nonessential amino acids (Millipore), 2 mmol/L GlutaMAX (Millipore), penicillin-streptomycin (Millipore), 20 ng/mL human LIF (Millipore), 8 ng/mL bFGF (Peprotech), 50 μg/mL bovine serum albumin (BSA; Sigma), and the following cytokines and small molecules: 1 μmol/L PD0325901 (Selleck), 0.5 μmol/L SB590885 (Selleck), 1 μmol/L WH-4-023 (Selleck), 10 μmol/L Y-27632 (Selleck), and 20 ng/mL activin A (PeproTech). TSCs were cultured in TSC medium (TSC basal medium supplemented with 2 μmol/L CHIR99021 (Selleck), 0.5 μmol/L A83-01 (Sigma), 1 μmol/L SB431542 (Selleck), 0.8 mmol/L valproic acid (VPA; Sigma), and 50 ng/mL EGF (Peprotech)). ExEnd cells were cultured in NACL medium (NACL basal medium supplemented with 100 ng/mL activin A (PeproTech), 10 ng/mL human LIF (Millipore), and 3 μmol/L CHIR99021 (Selleck)). The components of the TSC basal medium and NACL basal medium are shown below:

TSC basal medium: DMEM/F-12, GlutaMAX (Thermo Fisher) supplemented with 0.3% BSA (Sigma), 0.2% FBS (Thermo Fisher), 1% ITS-X supplement (Thermo Fisher), 0.1 mmol/L 2-mercaptoethanol (Thermo Fisher), 0.5% penicillin-streptomycin (Thermo Fisher), and 1.5 μg/mL l-ascorbic acid (Sigma). NACL basal medium: 1:1 mixture of DMEM/F-12 (Thermo Fisher) and neurobasal medium (Thermo Fisher), supplemented with 2 mmol/L GlutaMAX (Thermo Fisher), 0.1 mmol/L 2-mercaptoethanol (Thermo Fisher), 0.5% N2 supplement (Thermo Fisher), 1% B27 supplement (Thermo Fisher), and 1% penicillin-streptomycin (Thermo Fisher).

### Generation of human blastoids

For the generation of human blastoids, primed-to-naïve transition was performed to obtain the day 8 reset cells. Briefly, primed hESCs were dissociated into single cells and seeded on iMEFs with conventional hESC medium supplemented with Y-27632 (Selleck). The next day, the medium was switched to 5iLAF medium for 7 days. Then, the transitioning intermediate cells were dissociated by Accutase and counted and subjected to blastoid induction by seeding onto a 24-well AggreWell plate (Stem Cell Technologies). On day 6 of aggregation, blastoids were collected and evaluated. We used 5 × 10^4^, 7 × 10^4^, or 9 × 10^4^ starting cells for optimization of blastoid generation. To identify the optimal blastoid induction medium, we prepared the following seven groups: medium for Group 1, IVC1:NACL basal:TSC basal = 2:1:1; Group 2, IVC1:NACL basal:TSC basal = 4:1:1; Group 3, IVC1:NACL basal:TSC basal = 1:1:1; Group 4, IVC1:NACL basal:TSC basal = 0:1:1; Group 5, TSC basal:5Ilaf  =  1:1; Group 6, TSC basal:5iLAF = 2:1; and Group 7, TSC basal:5iLAF = 4:1.

IVC1 medium contained advanced DMEM/F-12 (Thermo Fisher), 1% ITS-X supplement (Thermo Fisher), 2 mmol/L l-glutamine (Thermo Fisher), 0.5% penicillin-streptomycin (Thermo Fisher), 20% FBS (Thermo Fisher), 25 μmol/L N-acetyl-l-cysteine (Sigma), 8 nmol/L β-estradiol (Sigma), and 200 ng/mL progesterone (Sigma).

For Groups 1–4, the mixing medium was supplemented with 2 μmol/L CHIR99021 (Selleck), 0.5 μmol/L A83-01 (Sigma), 1 μmol/L SB431542 (Selleck), 0.8 mmol/L valproic acid (VPA; Sigma), 50 ng/mL EGF (Peprotech), and 10 ng/mL BMP4 (Peprotech). For Groups 5–7, TSC basal medium was supplemented with 2 μmol/L CHIR99021 (Selleck), 0.5 μmol/L A83-01 (Sigma), 1 μmol/L SB431542 (Selleck), 0.8 mmol/L valproic acid (VPA; Sigma), and 50 ng/mL EGF (Peprotech).

### Derivation of stem cell lines from human blastoids

For the derivation of the b-pESCs, nESCs, and ExEnd cell lines, individual human blastoids were transferred onto iMEFs using a mouth pipette and cultured in conventional hESC medium, 5iLAF medium, or NACL medium for 5–7 days. The expanded outgrowths were then dissociated with 0.5 mmol/L EDTA or Accutase and seeded onto freshly prepared MEFs.

For the derivation of TSCs, TE-like cells were mechanically isolated using a glass microblade and were then transferred onto iMEFs in TSC medium. Cells were passaged with TryplE Express every 4–6 days.

### *In vitro* 2D attachment of human blastoids

*In vitro* 2D attachment of blastoids was performed as previously reported for human blastocysts (Shahbazi et al., 2016). Briefly, individual human blastoids were collected by mouth pipette and seeded into a four-well plate precoated with IVC1 medium for 2 days. Then, the medium was switched to IVC2 medium (advanced DMEM/F-12 (Thermo Fisher), 1% ITS-X supplement (Thermo Fisher), 2 mmol/L l-glutamine (Thermo Fisher), 0.5% penicillin-streptomycin (Thermo Fisher), 30% knockout serum replacement (KSR, Thermo Fisher), 25 μmol/L N-acetyl-l-cysteine (Sigma), 8 nmol/L β-estradiol (Sigma), and 200 ng/mL progesterone (Sigma)) for another 2 days. The attached blastoids were then fixed in PBS containing 4% paraformaldehyde (PFA, Sigma-Aldrich), and the supernatants were collected for the hCG test, following the instructions.

### *In vitro* 3D culture of human blastoids

*In vitro* 3D culture of blastoids was performed as previously reported for human blastocysts (Xiang et al., 2020). Briefly, individual human blastoids were collected by mouth pipette, transferred into a 96-well plate (corning #3474), and cultured in mIVC1 [IVC1 supplemented with 0.22% (*v*/*v*) sodium lactate (L7900, Sigma-Aldrich), 1 mmol/L sodium pyruvate (P4562, Sigma-Aldrich), and 10 μmol/L Y-27632 (S1049, Selleck)] for 2 days. Then, half of the medium was changed by mIVC2 [IVC2 supplemented with 0.22% (*v*/*v*) sodium lactate (L7900, Sigma-Aldrich), 1 mmol/L sodium pyruvate (P4562, Sigma-Aldrich), and 10 μmol/L Y-27632 (S1049, Selleck)] for 24 h. The blastoids were transferred into mICV2 containing 10% Matrigel on the next day. Half of the medium was changed every day thereafter. mIVC1 and mIVC2 were pre-equilibrated in the incubator for at least 6 h before use. The blastoid culturing conditions were as follows: 37.2°C, 6% CO_2_, and 5% O_2_.

### Frozen sectioning and immunofluorescence staining

For frozen sections, blastoids were fixed with 4% PFA (Sigma-Aldrich), washed at least three times with PBS containing 0.05% BSA, dehydrated twice with 15% sucrose for 3 min, and embedded in OCT. Embedded blastoids were sectioned by a Leica frozen slicer at a thickness of 10–12 μm. Before staining, the slides were washed with PBS to clear the remaining OCT.

For immunostaining, PSCs or blastoids were fixed in PBS containing 4% PFA (Sigma-Aldrich) overnight at 4°C, permeabilized in PBS containing 0.05% Triton X-100 for 30 min at room temperature, and incubated with blocking buffer (PBS containing 4% BSA) for 30 min. After incubation, primary antibodies were incubated overnight at 4°C followed by secondary antibodies at room temperature for 3–6 h. Nuclei were stained with 4’,6-diamidino-2-phenylindole (1:10,000; Sigma-Aldrich) in blocking buffer for 0.5–1 h. Images were taken using a Zeiss LSM880 Microsystem.

### RT-qPCR analysis

Total RNA was isolated from cells using TRIzol (Invitrogen). cDNA was synthesized using All-In-One RT MasterMix (ABM, G490) following the manufacturer’s instructions and amplified with ChamQ SYBR qPCR Master Mix (Vazyme) on a 7500 Fast Real-Time PCR system (Thermo Fisher). The GAPDH expression level was used as an internal normalization control. All statistical analyses and graphic illustrations were performed with GraphPad Prism (GraphPad Software, Inc.).

### Bulk RNA sequencing

Total RNA was isolated from cells using TRIzol (Invitrogen). A KAPA Stranded mRNA-Seq Kit (KAPA) was used following the manufacturer’s instructions. Adapters were offered by a TruSeq Library Prep Pooling kit (Illumina). Paired-end 150-bp sequencing was further performed on a NovaSeq 6000 (Illumina) at Berry Genomics Corporation.

### Single-cell RNA-seq data processing and integration

The 10× Genomics single-cell data were preprocessed using the Cell Ranger pipeline (v.4.0.0) with default parameters to generate the expression matrix. For quality control, all cutoffs were determined after investigating the distributions of each variable. Cells with a low or high number of expressed genes (nFeature), extremely high counts (nCount), or a high percentage of mitochondrial genes (pctMT) were discarded. The following thresholds were applied to retain cells: 2,000 < nFeature < 7,500; 20,000 < nCount < 150,000; and pctMT < 7.5. DoubletFinder was used to detect doublets (v.2.0.3). After quality control, 1,190 cells remained in the sample.

Previously published single-cell datasets (Petropoulos et al., 2016; Liu et al., 2021; Sozen et al., 2021; Yu et al., 2021) were integrated with our Blastoid datasets. Petropoulos’s 1,529 cells were filtered for blastocyst cells, removing the pre-blastocyst stages to leave 1,096 E5–E7 EPI, TE, and PE cells. To correct for technical differences and to perform an integrated analysis, we utilized the Seurat v.3 integration technique (v.3.2.3) (Stuart et al., 2019) and followed the official protocols provided by Satija Lab to integrate the different datasets. In brief, the functions NormalizeData (with default settings) and FindVariableFeatures (using 2000 features) were applied to the datasets separately, and then, the functions FindIntegrationAnchors (using 30 dimensions) and IntegrateData (using common genes) were applied to integrate the datasets. The resolution for cell clustering is 0.5.

### Gene signature scoring of single-cell RNA-seq and bulk RNA-seq samples

Scores of the gene signatures (EPI, TE, and PE) of single-cell RNA-seq were calculated with the AddModuleScore function in Seurat. For score calculation of the different gene signatures in the bulk RNA-seq samples, the expression range value (max—min) for each gene across all samples was first computed. Then, the scores of each gene of the gene set across all samples were computed by the formula: (gene expression—min)/(max—min), obtaining scaled gene expression ranging from 0 to 1. Finally, the sample score of the gene signatures was the mean expression of all the gene scores per sample. The primed, naïve, TE, EPI, and PE gene sets were obtained from Mackinlay et al., (2021) and Petropoulos et al., (2016).

### Quantification and statistical analyses

For immunostaining, *n* ≥ 3 biologically independent replicates were included. For bulk RNA-seq data of stem cell lines derived from human blastoids, *n* = 2 biological replicates were obtained for each sample. For 10× Genomics scRNA-seq data, libraries were generated from day 6 blastoids (*n* = 1). The number of cells used for downstream analysis was 1,190. Statistical analyses and graphics were carried out with GraphPad Prism 7 software. Detailed information can be found in specific parts of the Methods section and tables.

## Supplementary Material

pwac041_suppl_Supplementary_Materials

pwac041_suppl_Supplementary_Table_S1

pwac041_suppl_Supplementary_Table_S2

## Data Availability

The RNA-Seq data generated during this study are available at GEO: GSE200935. All the experimental materials generated in this study are available from the corresponding authors upon reasonable request.

## Abbreviations

EPI: epiblast
EPSCs: extended pluripotent stem cells
ESCs: embryonic stem cells
EVT: extravillous cytotrophoblast
ExEnd: extraembryonic endoderm
hCG: human chorionic gonadotrophin
hEPSCs: human extended pluripotent stem cell
hESCs: human embryonic stem cells
hPSCs: human pluripotent stem cells
ICM: inner cell mass
nESCs: naive embryonic stem cells
pESCs: primed embryonic stem cells
PrE: primitive endoderm
scRNA-seq: single-cell RNA sequencing
ST: syncytiotrophoblast
TE: trophectoderm
TSCs: trophoblast stem cells
TSNE: *t*-distributed stochastic neighbor embedding
UMAP: uniform manifold approximation and projection
ZGA: zygotic genome activation

## Code availability

Codes are available from the corresponding authors upon reasonable request.
