# Supplementary material for: Modeling human pregastrulation development by 3D culture of blastoids generated from primed-to-naïve transitioning intermediates
Source: Protein Cell. 2022 Sep 20;14(5):337–49. doi: 10.1093/procel/pwac041 (PMC10166166; doi:10.1093/procel/pwac041)
Supplement: pwac041_suppl_Supplementary_Materials [file pwac041_suppl_supplementary_materials.pdf]

Supplemental Information

Figure S1, related to Figure 1.

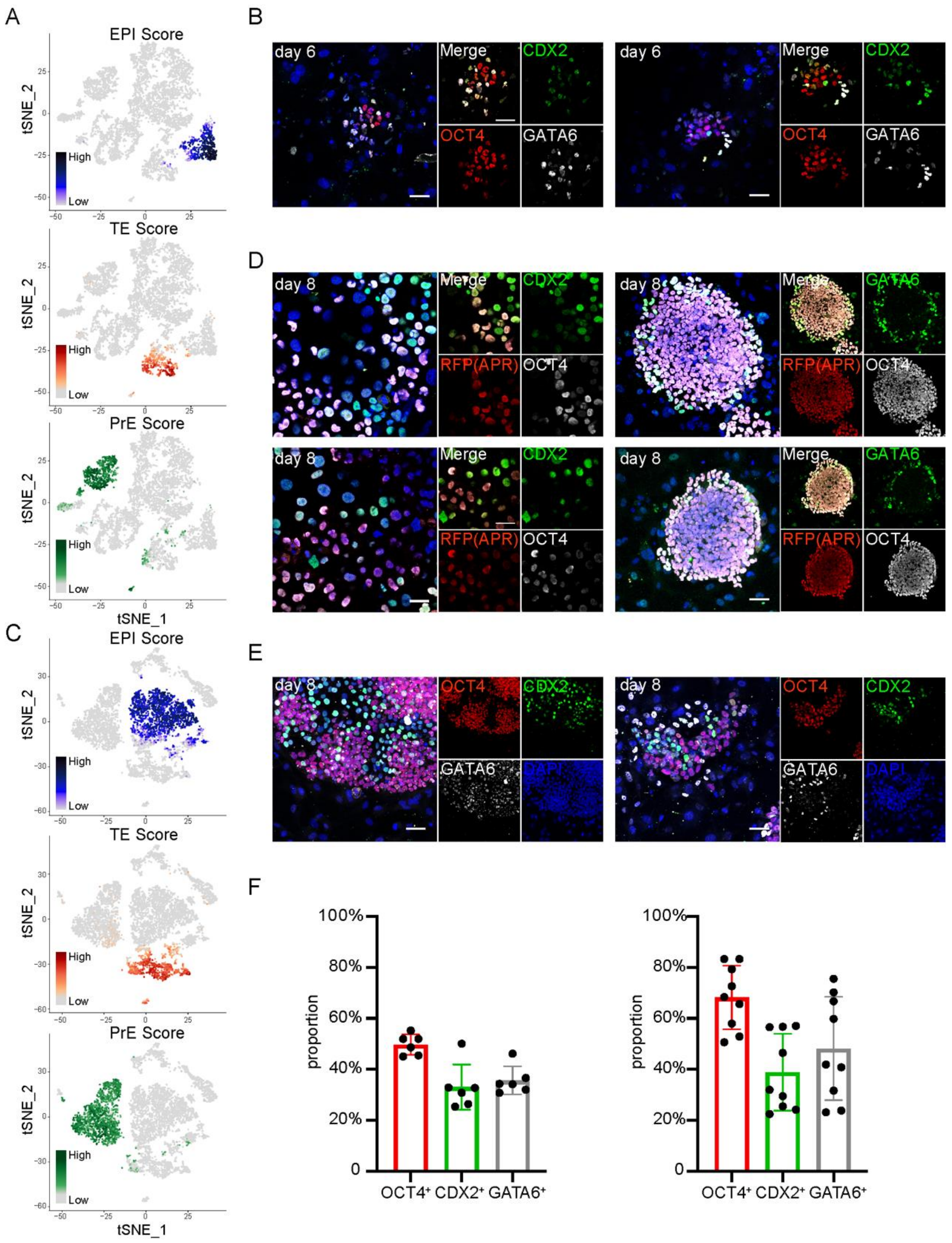

Figure S1, related to Figure 1.

Appearance of EPI, TE and PrE signatures in the day 6- and day 8-intermediate populations of the primed-to-naïve transition.

(A) Per-cell expression scoring analysis on tSNE showing the presence of EPI-like, TE-like and PrE-like populations in the transitioning intermediate cells on day 6 of the primed-to-naïve transition process.

(B) Representative images of co-immunostainings of OCT4, CDX2 and GATA6 in the day 6-intermediate cells of the primed-to-naïve transition process. Scale bars, 50 μm.

(C) Per-cell expression scoring analysis on tSNE showing the presence of EPI-like, TE-like and PrE-like populations in the transitioning intermediate cells on day 8 of the primed-to-naïve transition process.

(D) Representative images of co-immunostainings of OCT4, CDX2 and GATA6 in the day 8-intermediate cells during the naïve pluripotency resetting from H9 pESCs engineered with the *ALPG*-promoter-RFP (APR) reporter. Scale bars, 50 μm.

(E) Representative images of co-immunostainings of OCT4, CDX2 and GATA6 in the day 8-intermediate cells during the naïve pluripotency conversion from H9 pESCs. Scale bars, 50 μm.

(F) Quantification of cells with EPI, TE and PrE signatures respectively in the resetting cells on day 6 (left) and day 8 (right) of the primed-to-naïve transition shown in Figure S1B and S1D.

Figure S2, related to Figure 1.

A

group	cell number	size	cavity	group	cell number	size	cavity	group	cell number	size	cavity
1	5w	+	-	8	7w	+	√	15	9w	++	-
2	5w	+	-	9	7w	+	√	16	9w	++	√
3	5w	+	-	10	7w	+	-	17	9w	++	√
4	5w	+	-	11	7w	+	-	18	9w	++	-
5	5w	-	-	12	7w	+	-	19	9w	++	-
6	5w	-	-	13	7w	+	-	20	9w	++	-
7	5w	-	-	14	7w	+	-	21	9w	++	-

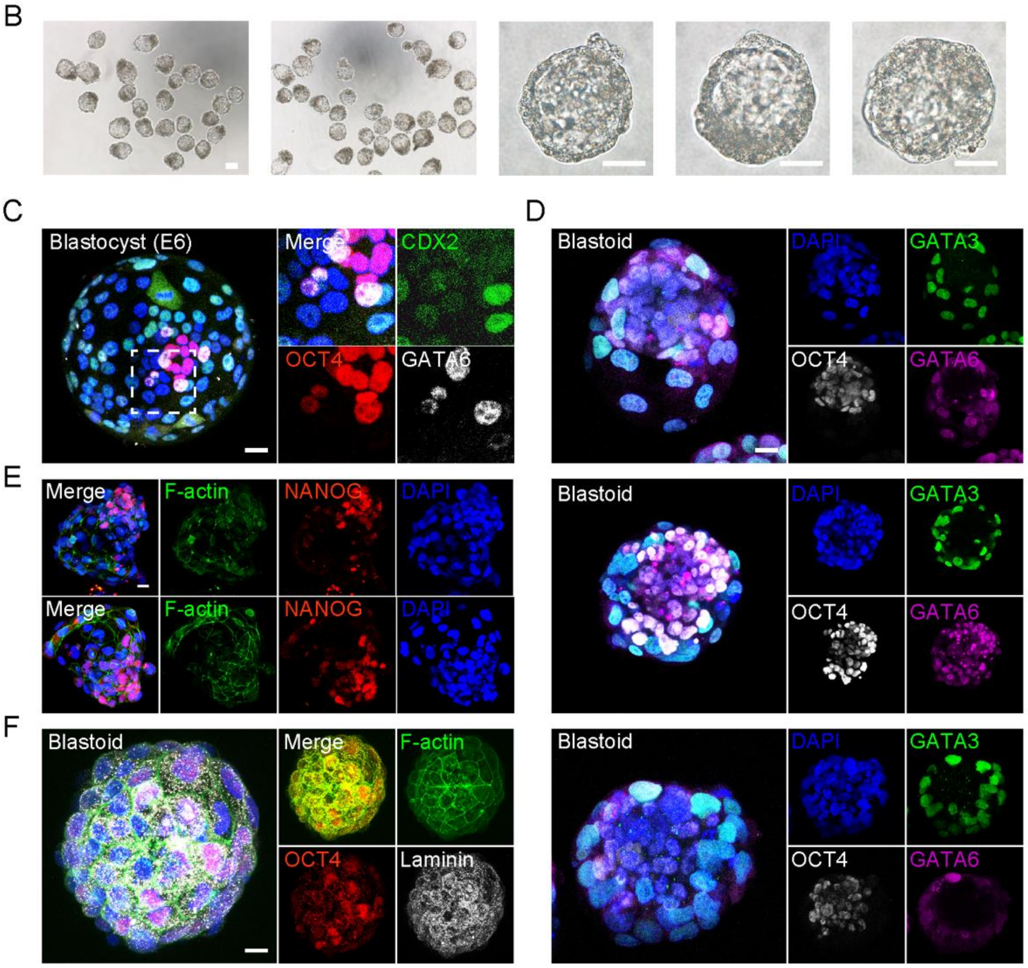

Figure S2, related to Figure 1.

Optimization and assessment of the blastoids generated from the day 8-intermediate cells of the primed-to-na ïve transition process.

(A) Summary of human blastoids generated from different starting cell numbers (5w, 7w, 9w, respectively) under different induction conditions (group 1-24). comparable size: +, over size: ++, -: fail to aggregation, √: succeed to aggregation and form cavity.

(B) Representative phase-contrast images of the blastoids. Scale bar, 100 μm.

(C) Representative images of co-immunostainings of OCT4, CDX2 and GATA6 in a blastocyst (E6). Scale bars, 20 μm.

(D) Representative images of co-immunostainings of OCT4, GATA3 and GATA6 in a blastoid. Scale bars, 20 μm.

(E) Representative images of co-immunostainings of F-actin and NANOG in a blastoid. Scale bars, 20 μm.

(F) Representative images of co-immunostainings of F-actin, OCT4 and Laminin in a blastoid. Scale bars, 20 μm.

Figure S3, related to Figure 2.

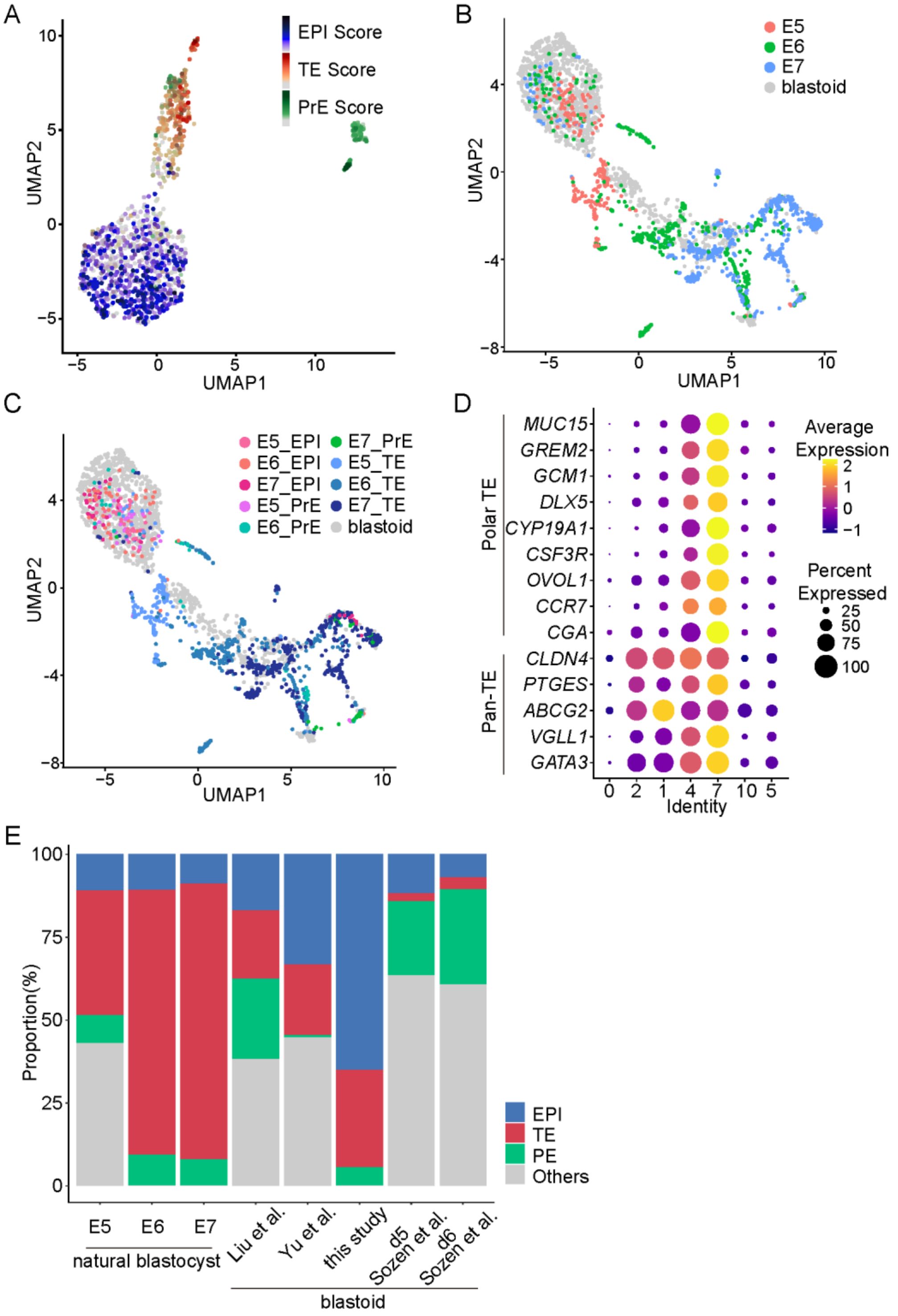

Figure S3, related to Figure 2.

scRNA-seq analysis of the blastoids generated from day 8-intermediate cells of the primed-to-naïve transition.

(A) Per-cell expression scoring analysis for EPI, TE, and PrE signatures on UMAP of the blastoid scRNA-seq dataset.

(B) UAMP plots of the integrated scRNA-seq datasets (Petropoulos et al., 2016) of blastoids (grey) and pre-implantation embryos in Figure 2A at E5 (red), E6 (green), and E7 (blue).

(C) UAMP plots highlighting cells of the blastoids (grey) and cells of pre-implantation embryos in Figure 2A at E5-E7 with EPI, TE and PrE signatures (labeled with different colors).

(D) Dot plot indicating the expression of trophectoderm (TE) markers.

(E) Bar plot showing lineages composition of natural blastocyst and blastoids. Others represent pre-lineage or undefined clusters.

Figure S4, related to Figure 2.

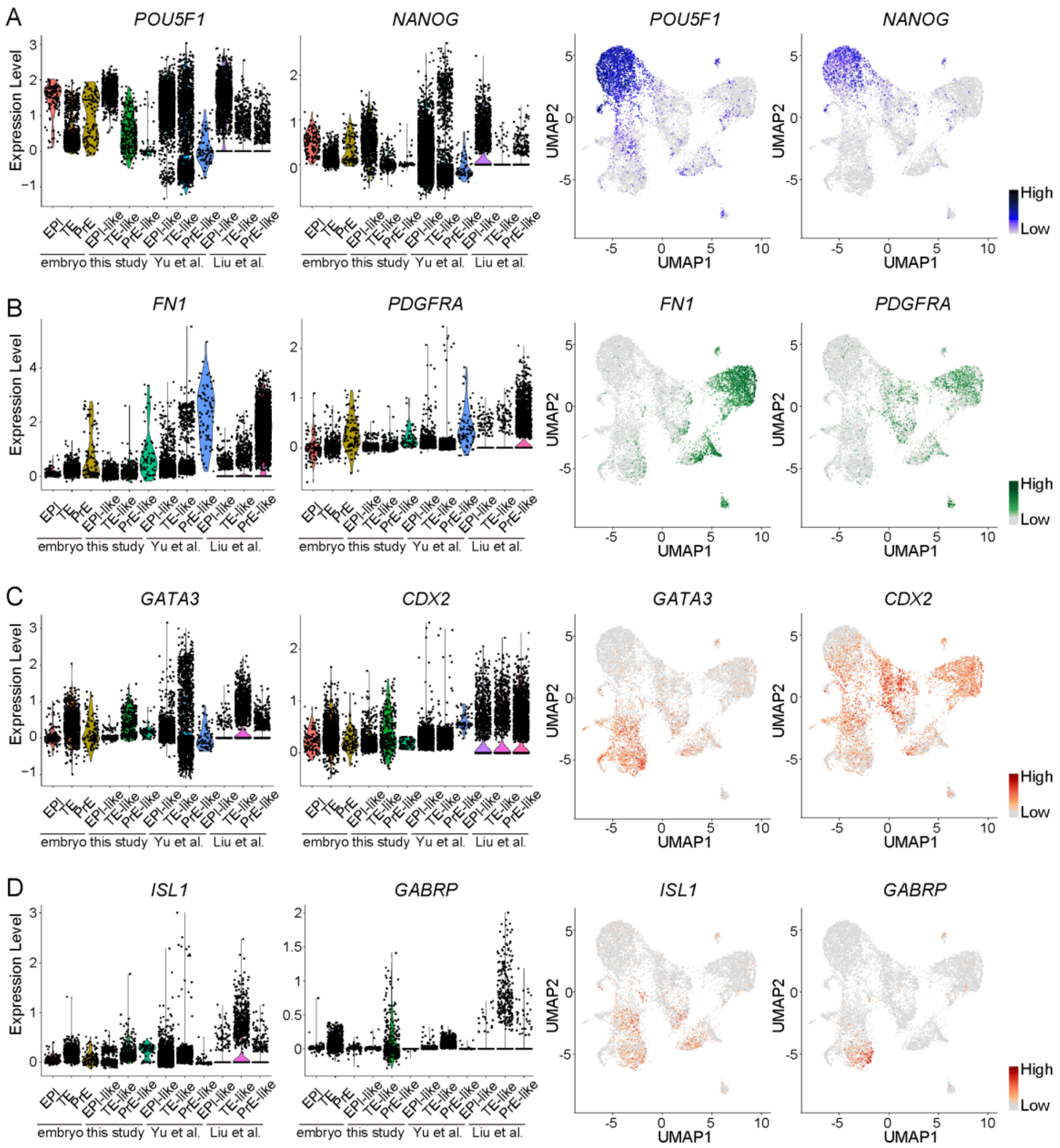

Figure S4, related to Figure 2.

Specific genes expression in natural blastocyst and blastoids

(A-D) Violin plots and UMAP plotsshowing the expression levels of EPI-specific genes (*POU5F1*, *NANOG*), TE-specific genes (*GATA3*, *CDX2*), PrE-specific genes (*FN1*, *PDGFRA*) and amnion-specific genes (*ISL*1, *GABRP*) in natural blastocyst and blastoids.

Figure S5 related to Figure 3.

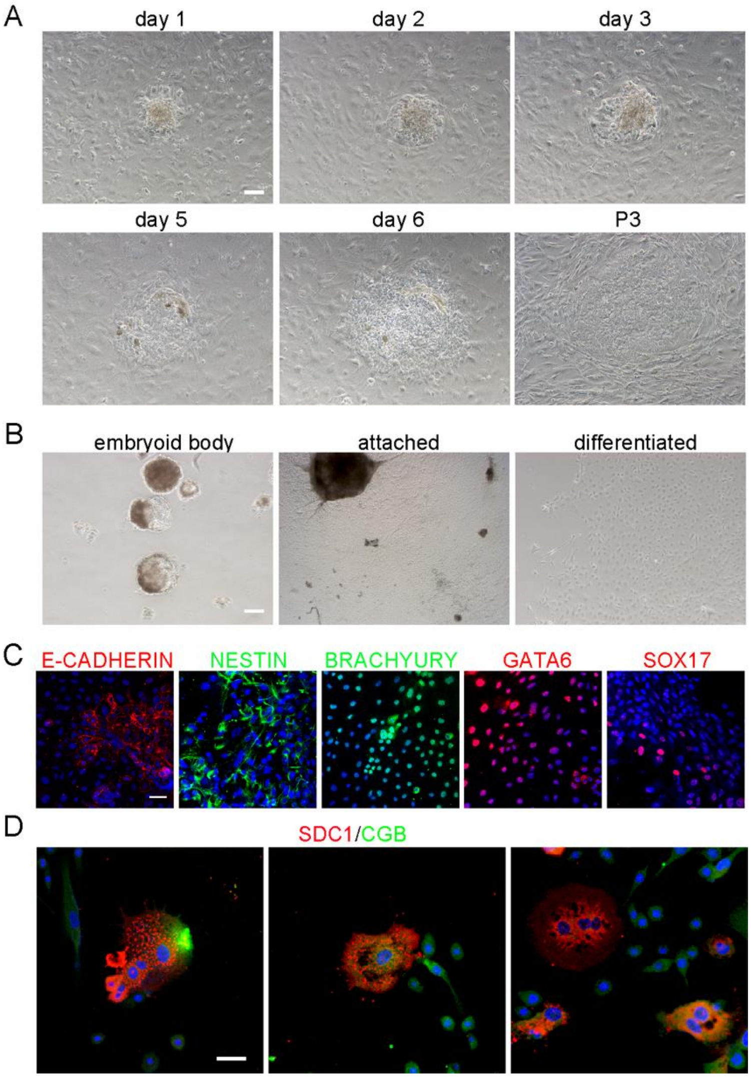

Figure S5, related to Figure 3.

Derivation and differentiation of different stem cell lines from the blastoids.

(A) Representative bright-field images of outgrowths (from days 1 to day 6) and a derivative ESC colony during the derivation of b-pESCs. Scale bars, 100 μm.

(B) Representative bright-field images showing differentiation of b-pESCs by embryoid body formation. Scale bars, 100 μm.

(C) Immunofluorescence images of representative ectoderm (E-CADHERIN, NESTIN), mesoderm (BRACHYURY) and endoderm (GATA6 and SOX17) markers in the cells differentiated from b-pESCs by embryoid body formation shown in Figure S4B. Scale bars, 50 μm.

(D) Immunofluorescence images of SDC1 and CGB in ST cells differentiated from b-TSCs. Scale bars, 20 μm.

Figure S6, related to Figure 4.

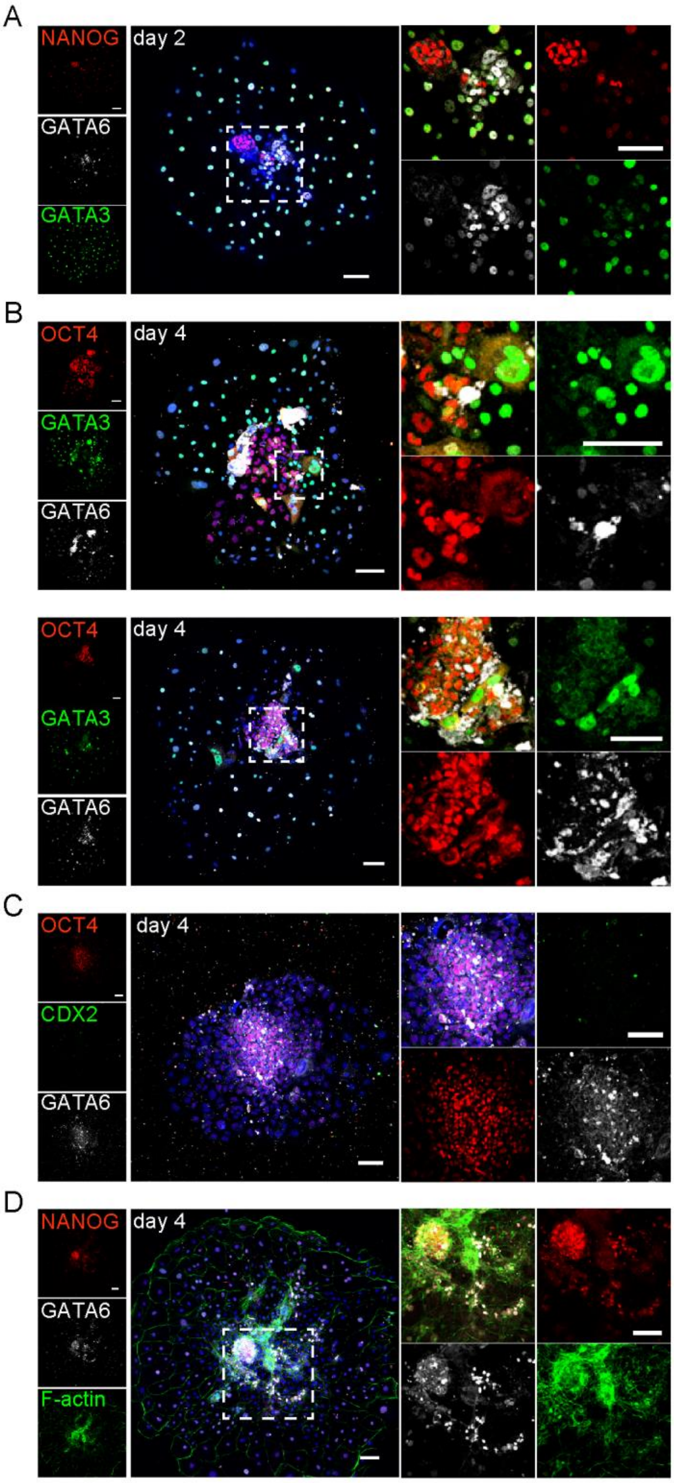

Figure S6, related to Figure 4.

2D Culture of human blastoids modelling peri-implantation stages.

(A) Representative images of co-immunostaining of NANOG, GATA3 and GATA6 in a day 2 attached human blastoid, Scale bars, 100 μm.

(B) Representative images of co-immunostaining of OCT4, GATA3 and GATA6 in a day 4-attached human blastoid, Scale bars, 100 μm.

(C) Representative images of co-immunostaining of OCT4, CDX2 and GATA6 in a day 4-attached human blastoid, Scale bars, 100 μm.

(D) Representative images of co-immunostaining of NANOG, F-actin and GATA6 in a day 4-attached human blastoid, Scale bars, 100 μm.

Figure S7, related to Figure 5.

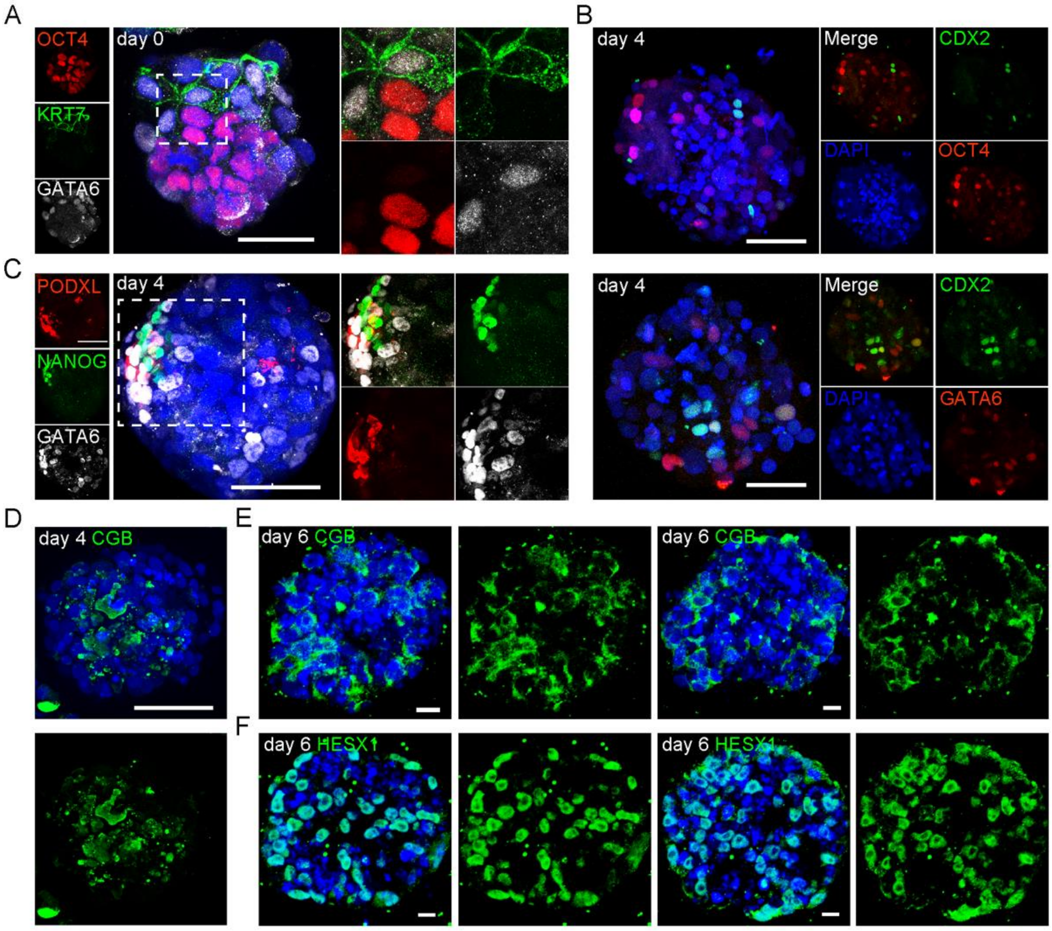

Figure S7, related to Figure 5.

3D Culture of human blastoids modelling human post-implantation development.

(A) Representative images of co-immunostaining of OCT4, KRT7 and GATA6 in a blastoids cultured in the 3D system on day 0, Scale bars, 50 μm.

(B) Representative images of co-immunostaining of CDX2, OCT4 or GATA6 in a blastoid cultured in the 3D system on day 4, Scale bars, 100 μm.

(C) Representative images of co-immunostaining of NANOG, GATA6 and PODXL in a blastoids cultured in the 3D system on day 4, Scale bars, 100 μm.

(D) Representative images of CGB immunostaining in a blastoid cultured in the 3D system on day 4, Scale bars, 50 μm.

(E) Representative frozen section images of CGB immunostaining in a blastoid cultured in the 3D system on day 6, Scale bars, 20 μm.

(F) Representative frozen section images of HESX1 immunostaining in a blastoid cultured in the 3D system on day 6, Scale bars, 20 μm.
